# Factors Affecting Workplace Bullying Perpetration Among Experienced Intensive Care Unit Nurses: A Secondary Analysis Focusing on Narcissistic Personality, Perfectionistic Self-Presentation, and Mentalization

**DOI:** 10.1155/jonm/9156280

**Published:** 2025-09-18

**Authors:** Sun Joo Jang, Jeehae Chung, Haeyoung Lee

**Affiliations:** ^1^College of Nursing & The Research Institute of Nursing Science, Seoul National University, Seoul 03080, Republic of Korea; ^2^College of Nursing, The Catholic University of Korea, Seoul 06591, Republic of Korea; ^3^Red Cross College of Nursing, Chung-Ang University, Seoul 06974, Republic of Korea

**Keywords:** intensive care unit, mentalization, narcissism, personality, workplace bullying

## Abstract

**Aim:** Workplace bullying is a critical issue faced by nurses globally. This study aimed to examine factors influencing workplace bullying perpetration among experienced intensive care unit nurses in South Korea, while controlling for organizational culture. Specifically, it investigated how narcissistic personality, perfectionistic self-presentation, and mentalization affected bullying perpetration.

**Methods:** In this cross-sectional secondary analysis, data were collected between July 1 and August 2, 2022; we analyzed data from 287 nurses who fit the inclusion/exclusion criteria. We performed multiple regression analyses to identify factors affecting workplace bullying perpetration.

**Results:** The findings showed that narcissistic grandiosity (*β* = 0.21, *p*=0.017), narcissistic vulnerability (*β* = 0.21, *p*=0.031), mentalization (*β* = −0.19, *p*=0.001), and previous workplace bullying victimization (*β* = 0.18, *p*=0.002) were significant predictors. The regression model explained 30.1% of the variance in workplace bullying perpetration.

**Conclusion:** Importantly, narcissistic personality, perfectionistic self-presentation, and mentalization were significantly associated with workplace bullying perpetration among nurses. These findings suggest that understanding the links between individual personality traits and bullying behavior may provide useful insights for developing supportive interventions, such as programs aimed at enhancing mentalization. While the study did not establish causal relationships, the associations highlight potential areas for further research and practice. In addition, organizational efforts to address workplace bullying more broadly are essential to prevent its occurrence and reduce its negative impact.

## 1. Introduction

Workplace bullying is a significant issue faced by nurses worldwide, affecting not only individual nurses but also hospital organizations [1, 2]. It is characterized by repeated and malicious actions targeting individuals or groups [3]. Workplace bullying causes physical distress such as headaches, fatigue, sleep disorders, and gastrointestinal complaints, as well as emotional distress such as burnout, emotional exhaustion, depression, anxiety, post-traumatic stress disorder, and suicidal ideations [2, 4–6]. Moreover, it extends beyond individual harm, negatively affecting the overall functioning of nursing organizations by compromising teamwork, communication, and concentration at work. This leads to job dissatisfaction, increased turnover rates, and knowledge hiding behaviors, ultimately resulting in substantial costs for healthcare institutions [4, 7–9]. Given that nurses play a crucial role in patient care, workplace bullying ultimately affects the quality of nursing care and can lead to severe issues that threaten patient safety [10]. Despite the serious consequences of workplace bullying, a recent scoping review found that nurses are frequently dissatisfied with their organization's handling of reported incidents. Moreover, multiple individual and organizational factors hinder formal reporting, with few effective strategies identified to promote it [11].

The intensive care unit (ICU) is a highly complex and demanding environment in which stressful situations frequently occur [12]. In such settings, interactions among nurses are often negatively affected, leading to conflicts and discord that may eventually result in workplace bullying [13]. ICU nurses are particularly susceptible to horizontal violence related to nursing tasks among colleagues [14].

Moreover, workplace bullying remains a persistent and unresolved issue, necessitating a deeper understanding of its contributing factors [15]. Previous studies suggest that workplace bullying is associated with organizational-level factors, such as workload, leadership styles, highly stressful working environments, and organizational cultures that condone bullying [16, 17]. Workplace bullying has also been examined from the perspective of individual-level factors, such as demographic characteristics and personality traits [2, 4]. Given that workplace bullying emerges from the interaction between institutional and individual factors, a comprehensive approach is essential to fully understand this phenomenon [18].

In poorly managed, high-stress environments, perpetrators often exhibit workaholic tendencies and justify their actions, expecting favorable treatment [19]. Additionally, perpetrators often exhibit negative personality traits such as narcissism, manipulativeness, and psychopathy and may believe that the victim deserved to be bullied [20, 21]. In a study that examined the influencing factors of workplace bullying among hospital nurses, perpetrator behavior was influenced by emotional intelligence, meritocracy beliefs, and personal bullying experiences [22]. Moreover, according to a review on workplace bullying among nurses, perpetrators were identified as individuals who occupy a higher position within the hierarchy. They exhibited a strong desire to dominate, often instigated conflict to exert power through violence, and continued bullying without fear of repercussions [17].

The parent study controlled for organizational culture and focused specifically on individual factors by investigating the effects of personality traits and mentalization on workplace bullying among ICU nurses [20]. It analyzed workplace bullying by addressing the perspectives of both the perpetrator and the victim. It identified that narcissistic vulnerability, perfectionistic self-presentation, and mentalization were significant influencing factors of workplace bullying from the victim's perspective. In contrast, a dark personality, perfectionistic self-presentation, and mentalization were key influencing factors from the perpetrator's perspective [20].

Pathological narcissism is characterized by self-centeredness, a lack of empathy, entitlement, exploitative or arrogant behavior (narcissistic grandiosity), and contingent self-esteem and entitlement rage (narcissistic vulnerability) [23, 24]. Pathological narcissists often display an inflated sense of their abilities and a resistance to criticism, resulting in conflicts with colleagues and workplace bullying [25]. Perfectionistic self-presentation involves concealing imperfections and striving to appear flawless, which can lead to blaming or attacking others, especially in performance-driven organizations [26, 27]. Mentalization is the ability to understand one's own and others' emotions, and deficits in this ability can cause interpersonal problems and increase perpetrator behavior in the workplace [20, 28, 29]. This is particularly important in high-stress environments such as the ICU, where nurses with impaired mentalization are more likely to exhibit bullying behavior [28].

Understanding personality traits and their influence on perpetrator behavior is essential for developing practical and effective intervention strategies to address workplace bullying. Furthermore, less experienced nurses are at higher risk of being bullied [2]. Thus, it is essential to investigate perpetrator behavior among more professionally experienced nurses—particularly those with three or more years of employment, who are typically classified as experienced practitioners [30]. Therefore, this study analyzes the factors affecting workplace bullying perpetration, while controlling for organizational culture, among nurses with at least 3 years of work experience in an ICU at a tertiary hospital in South Korea. This study focuses on the relationships between narcissistic personality, perfectionistic self-presentation, and mentalization among individual nurses.

## 2. Materials and Methods

### 2.1. Study Design

We employed a cross-sectional secondary analysis.

### 2.2. Participants

The inclusion criteria encompassed nurses who (1) were working in the ICU of a tertiary hospital, (2) had at least 3 years of experience, and (3) were providing care to patients either directly or indirectly. Based on previous studies indicating a high degree of workplace bullying perpetration in the ICU [13, 14], nurses not involved or indirectly involved in patient care (i.e., education nurses and administrative nurses) were excluded. The exclusion criteria were (1) unit managers and (2) individuals not affiliated with the nursing department (i.e., research nurses affiliated with medical departments). Nurse managers were excluded to specifically focus on peer-to-peer (horizontal) workplace bullying among nursing staff, which involves distinct interpersonal dynamics.

### 2.3. Data Collection and Ethical Considerations

Data for the parent study [20] were collected between July 1 and August 2, 2022. Data for the parent study were collected via an online survey administered through a Google Form link, which was distributed using an open-call approach. Participants were advised not to submit duplicate responses. The survey was promoted on online platforms primarily used by nurses, including tertiary hospital groupware bulletin boards. Of the 416 responses included in the parent study, 287 were selected for this secondary data analysis based on the inclusion and exclusion criteria defined for the current study. We estimated the required sample size for multiple regression analyses using G∗power 3.1.9.7 with a two-tailed significance level of 0.05, power of 0.99, an effect size of *f*^2^ = 0.15 (medium). There were 14 predictors, nine of them comprising participants' general and work-related characteristics (sex, marital status, religion, educational level, total working years, position, subjective health status, antibullying education, and previous workplace bullying victimization) and five main variables (narcissistic grandiosity, narcissistic vulnerability, perfectionistic self-presentation, mentalization, and organizational culture). The minimum required sample size was calculated to be 251 persons, implying that the sample size of 287 persons was adequate. Before commencing the study, the institutional review board at the authors' affiliated institution granted an exemption from review, given that the study constituted a secondary data analysis (No. 1041078-20221106-HR-006, date of approval: December 16, 2022). Participants provided informed consent after reading a document explaining the study's purpose and details. They were assured of their right to withdraw at any time, thus guaranteeing voluntary participation. The survey did not collect any personally identifiable information, such as names or affiliations, ensuring that participant anonymity was maintained by third parties, including the researchers.

### 2.4. Instruments

#### 2.4.1. General and Work-Related Characteristics

Based on previous research [13, 20], a self-report questionnaire was developed by the authors to obtain information on participants' general and work-related characteristics (sex, marital status, religion, educational level, age, total working years, position, subjective health status, antibullying education, and previous workplace bullying victimization).

#### 2.4.2. Narcissistic Personality

To measure narcissistic personality, we used the Korean version of the Pathological Narcissism Inventory, culturally adapted by Yang and Kwon [31] from the original instrument developed by Pincus et al. [32]. The Korean version comprises two subdomains (narcissistic grandiosity and narcissistic vulnerability) and 35 items in total. Each item is scored on a 6-point Likert-type scale (scoring range, 0–210 points), with higher scores being interpreted as more severe pathological narcissism. Cronbach's *α* was 0.95 (0.78–0.93) for the original instrument [32], 0.92 (0.85–0.92) for the Korean version [31], and 0.96 (0.91–0.95) in this study.

#### 2.4.3. Perfectionistic Self-Presentation

Perfectionistic self-presentation was evaluated using the Korean version [33] of the Perfectionistic Self-Presentation and Psychological Distress scale [34]. The original instrument comprises 27 total items across three subdomains (perfectionistic self-promotion, nondisplay of imperfection, and nondisclosure of imperfection); however, 8 items were deleted in the process of cultural adaptation [33]. Therefore, the Korean version comprises 19 items divided among the same three subdomains. Each item is scored on a 7-point Likert-type scale (1 point: “*not at all*,” 7 points: “*always*”; scoring range, 19–133 points), with higher scores indicating a stronger tendency toward perfectionistic self-presentation. Cronbach's *α* was 0.91–0.95 for the original instrument [33], 0.85 (0.75–0.88) for the Korean version [33], and 0.89 (0.76–0.90) in this study.

#### 2.4.4. Mentalization

The Korean version of the Mentalization Scale was used to measure mentalization. The scale was originally developed by Dimitrijević et al. [35] and was culturally adapted into Korean by Lee and Lee [36]. This instrument comprises 25 items in total across three subdomains (other-related mentalization: 11 items, self-related mentalization: 6 items, and motivation to mentalize: 8 items). Each item is scored on a 5-point Likert-type scale (1 point: “*completely incorrect*,” 5 points: “*completely correct*”; scoring range, 25–125 points). Cronbach's *α* was 0.84 (0.74–0.79) for the original instrument [35], 0.88 (0.74–0.84) for the Korean version [36], and 0.84 (0.74–0.84) in this study.

#### 2.4.5. Organizational Culture

The control variable of organizational culture was evaluated using the Positive Nursing Organizational Culture Measurement Tool [37]. This tool comprises 26 items across 4 subdomains (pursuit of common values: 7 items, forming organizational relationships based on trust: 8 items, fair management system: 4 items, and positive leadership of nursing unit manager: 7 items). Each item is scored on a 5-point Likert-type scale (1 point: “*strongly disagree*,” 5 points: “*strongly agree*”; scoring range, 26–130 points), with higher scores indicating that the respondent rated the culture at their organization more positively. Cronbach's *α* was 0.95 (0.83–0.95) at the time of development [37] and 0.96 (0.83–0.93) in this study.

#### 2.4.6. Workplace Bullying Perpetration

We evaluated workplace bullying perpetration from the perpetrator's perspective using the Negative Acts Questionnaire-Perpetrator, which is a modified version of The Negative Acts Questionnaire—Revised [38] with confirmed reliability and validity. It is a unidimensional self-report instrument comprising 22 items, each rated on a five-point Likert scale (1 = *strongly disagree* to 5 = *strongly agree*), with total scores ranging from 22 to 110. The tool assesses behaviors such as “Intimidation, such as finger-pointing, invasion of personal space, shoving, blocking someone's way.” Cronbach's *α* was 0.97 at the time of development and 0.96 in this study.

### 2.5. Statistical Analysis

SPSS Statistics program 26.0 Version (IBM CORP., Armonk, NY) was used for data analysis. To assess the normality of the variables, skewness and kurtosis were examined, and normality was determined according to the criteria suggested by Kline [39]. Differences in workplace bullying perpetration depending on the participants' general and work-related characteristics were analyzed using *t*-tests and one-way analysis of variance. Correlations between the main variables were analyzed using Pearson correlation coefficients. Furthermore, multiple regression analyses were conducted to identify the factors influencing workplace bullying perpetration. Variables that showed significant associations in the univariate analysis were entered into the models, with organizational culture additionally included as a covariate to control for its potential confounding effect. The normality of residuals was assessed using the Kolmogorov–Smirnov test applied to the standardized residuals of the regression model.

## 3. Results

### 3.1. Participants' General and Work-Related Characteristics

There were no missing values. Of the 287 participants, 270 were females (94.1%), 181 were unmarried (63.1%), 156 had no religion (54.4%), and 225 held an undergraduate degree as their highest level of education (78.4%). The mean age of the participants was 32.45 years (standard deviation 5.40 years), and the mean total experience was 7.70 years (standard deviation 4.30 years). The most common job title was staff nurse (241 persons, 84.0%), and most participants had a subjective health status of “good” (239 persons, 83.3%). Within the last year, 140 persons (48.8%) had received antibullying education, and 60 persons (20.9%) had experienced previous workplace bullying victimization (Table 1).

### 3.2. Differences in Workplace Bullying Perpetration Depending on General Characteristics and Work-Related Characteristics

Scores for workplace bullying (perpetrator aspect) exhibited no significant differences based on sex, marital status, educational attainment, job title, or recent antibullying education. Conversely, workplace bullying perpetration scores were higher for participants with a religion, poor subjective health status, and previous experience of workplace bullying victimization (Table 2).

### 3.3. Correlation Among Major Variables

Workplace bullying perpetration was positively correlated with narcissistic grandiosity (*r* = 0.42, *p* < 0.001), narcissistic vulnerability (*r* = 0.47, *p* < 0.001), and perfectionistic self-presentation (*r* = 0.15, *p*=0.012) but negatively correlated with mentalization (*r* = −0.31, *p* < 0.001) and organizational culture (*r* = −0.12, *p*=0.040; Table 3).

### 3.4. Influencing Factors of Workplace Bullying Perpetration

Using the enter method, we performed a multiple linear regression analysis. The variance inflation factors ranged between 1.09 and 3.83, exhibiting no multicollinearity between the independent variables. The Durbin–Watson index was 1.95 (du = 1.86, 4-du = 2.14), indicating independence of the error terms; the residuals were normally distributed (*Z* = 0.05, *p*=0.269), implying that the assumptions for the regression analysis were satisfied. The regression model, which controlled for organizational culture, explained 30.1% of the variance in workplace bullying perpetration. Significant influencing factors for workplace bullying perpetration included narcissistic grandiosity (*β* = 0.21, *p*=0.017), narcissistic vulnerability (*β* = 0.21, *p*=0.031), mentalization (*β* = −0.19, *p*=0.001), and previous workplace bullying victimization (*β* = 0.18, *p*=0.002). Specifically, workplace bullying perpetration scores were higher for participants with more severe narcissistic grandiosity and narcissistic vulnerability, poorer mentalization, and previous experience of workplace bullying victimization (Table 4).

## 4. Discussion

This study investigated the relationships between the influencing factors of workplace bullying perpetration, while controlling for organizational culture, among nurses who had been working for at least 3 years in the ICU of a tertiary hospital. Of these factors, we specifically focused on narcissistic personality, perfectionistic self-presentation, and mentalization.

Significant influencing factors for workplace bullying perpetration included narcissistic grandiosity, narcissistic vulnerability, mentalization, and previous workplace bullying victimization. Specifically, workplace bullying perpetration scores were higher for participants with more severe narcissistic grandiosity and narcissistic vulnerability, poorer mentalization, and previous experience of workplace bullying victimization.

We found that narcissistic grandiosity and narcissistic vulnerability were major influencing factors of workplace bullying perpetration, which aligns with previous research. Narcissism and aggression have been reported to be significantly related in a meta-analysis [40]. One recent systematic review analyzing the properties of workplace bullying perpetration also demonstrates that narcissistic traits were among the various personality traits of perpetrators [17]. Narcissism can be categorized as subclinical narcissism (normal narcissism), which occurs normally during development, and clinical narcissism (pathological narcissism), which leads to maladaptation to daily living [40]. The negative aspects of pathological narcissism are reported to be most apparent in interpersonal relationships [41]. Nurses with narcissistic personality traits are likely to experience interpersonal difficulties, including involvement in workplace bullying. Such narcissistic behaviors may negatively impact nurses' colleagues and organization [25]. Therefore, planning and providing interventions for active management, such as regulating emotions and self-esteem, are crucial for preventing narcissistic personality traits from perpetrating negative behavior [31, 42].

The finding that mentalization ability is negatively associated with workplace bullying perpetration is highly meaningful. Mentalization refers to the capacity to understand one's own and others' mental states; individuals with higher mentalization capacity are more likely to consider others' perspectives and refrain from aggressive behaviors [28]. Consequently, poor mentalization can result in a limited understanding of oneself and others, leading to interpersonal difficulties. In addition, the high work-related stress typical of ICU environments may further weaken nurses' mentalization abilities. Similarly, Pedditzi et al. reported that mentalization ability is closely related to bullying behavior, supporting the present findings [29]. Thus, nurses with poor mentalizing ability may be more likely to perpetrate bullying in the workplace. Therefore, it is necessary to develop and implement interventions to reduce work stress for nurses and help them maintain stable mentalizing abilities.

Finally, the experience of workplace bullying (victim) was found to be a significant influencing factor of workplace bullying perpetration. Notably, this experience was not a substantial factor in the parent study [19]. However, it demonstrated statistical significance when the analysis was restricted to nurses with at least 3 years of experience. These findings support the cyclical pattern identified by Vranjes et al., where victims and perpetrators interchange roles [43]. This demonstrates how previously victimized individuals may displace their suffering onto others or adopt aggressive behaviors as organizational survival strategies. According to a recent systematic review on the making and breaking of workplace bullying perpetration [19], when bullying is ignored, accepted, or uncriticized in an organization, many people adopt this as learned behavior. As these behaviors get repeated when people attempt to solve the problem of bullying themselves, previous victims also become perpetrators of workplace bullying in some cases. The fact that nurses with previous experience of workplace bullying victimization exhibited higher workplace bullying perpetration scores could indicate a perpetuating cycle of workplace bullying. This suggests that prevention of harm from workplace bullying during nurses' training could be important to break the cycle of workplace bullying. However, one related study found that more workplace bullying was reported after establishing or reinforcing these policies, and that workplace bullying perpetrators could become targets again [43]. Therefore, a multidimensional approach and close analysis of related factors are essential for establishing basic plans to stop workplace bullying. Taylor et al. [44] highlighted that studies on workplace incivility have ignored the unique relationship between dyad members as a contributing factor and focused predominantly on the individual characteristics and problems of the perpetrator and victim. Therefore, studies analyzing the bidirectional relationship between the perpetrator and victim, and the influencing factors of this relationship, warrant more attention. Islam and Chaudhary [6] reported that workplace friendship played a critical role in buffering the negative impact of workplace bullying on knowledge hiding. Building on this, fostering a workplace culture that encourages friendship among nurses may serve as a valuable strategy for reducing workplace bullying.

This study used data from nurses with at least 3 years of experience, a decision based on findings that experienced nurses often become perpetrators of workplace bullying [45], and the previous standard of categorizing nurses with at least 3 years of experience as “experienced nurses” [30]. In the parent study [20], which also included nurses with less than 3 years of experience, a dark personality, perfectionistic self-presentation, and mentalization were the main influencing factors of workplace bullying perpetration. However, in this study, narcissistic grandiosity, narcissistic vulnerability, mentalization, and experience of workplace bullying (victim) were identified as significant influencing factors. This demonstrated that the influencing factors of workplace bullying perpetration by ICU nurses differ depending on experience. Compared with other nursing units, ICU patients are typical in critical condition, requiring complex care and swift, accurate interventions [46]. This high-pressure environment may contribute to workplace bullying among ICU nurses, which often manifest as horizontal violence between peers [47, 48]. Moreover, workplace bullying exacerbates knowledge hiding among nurses [6]. Given that effective nursing care depends on information and knowledge sharing, this relationship may create a vicious cycle in which knowledge hiding undermines collaboration and subsequently contributes to the escalation of work-related bullying. Therefore, identifying the specific characteristics of work-related bullying in ICU settings and preventing its progression are essential to breaking this negative cycle. Additionally, according to a systematic review on workplace bullying perpetration [18], poor work design and stressful working conditions cause overwork and interpersonal conflict, lead to discord within teams, and increase expression of anger toward other people [18]. Conversely, individual personality traits may lead some individuals to exhibit negative behaviors even in resource-rich work environments, indicating that the availability of work resources alone cannot fully prevent such behaviors [18]. Therefore, when analyzing the influencing factors of workplace bullying perpetration, it is essential to analyze the effects from multiple angles, including work characteristics and working conditions.

As this study was a secondary analysis, there were limitations in including work-related variables for experienced ICU nurses with at least 3 years of experience. Future research should consider characteristics associated with nurses' experiences more precisely when analyzing influencing factors of workplace bullying perpetration. Furthermore, while numerous studies have examined how the work environment influences workplace bullying, there is a notable lack of research exploring whether perpetrators' negative behaviors alter the work environment [18]. Therefore, we propose that further studies should be conducted on the effects of bullying perpetration on the work environment.

This study has several limitations. First, because this was a secondary analysis, the research variables were limited to those in the parent study. As mentioned above, we propose that further studies on the various influencing factors of workplace bullying perpetration should carefully consider the work-related characteristics of ICU nurses, particularly those that might differ depending on experience. Second, because this study was based on cross-sectional data, we were unable to prove causal relationships between the main variables. Additionally, as the data were collected through self-report questionnaires, we cannot exclude the possibility that participants' responses could have been over- or underestimated. Third, because the study participants included only a small percentage of nurses working in the ICU of a tertiary hospital in South Korea, the generalizability of the findings is limited. Therefore, evidence for this study's results needs to be reinforced by conducting studies in more diverse settings.

## 5. Conclusions

This study confirmed that, among ICU nurses with at least 3 years of experience, higher workplace bullying perpetration scores were associated with greater levels of narcissistic grandiosity and narcissistic vulnerability, poorer mentalization, and prior experiences of workplace bullying (as victims). These findings suggest that educational opportunities should be provided to help nurses understand their own and other people's personality traits and maintain interpersonal relationships appropriately. Implementing intervention programs to strengthen mentalization is particularly important given its association with bullying perpetration. The finding that the experience of workplace bullying victimization was associated with higher perpetration scores indicates the need for strengthened preventative policies to protect nurses from becoming victims during their professional development. Organizations should consider active intervention strategies, as inadequate policies may contribute to cycles of workplace bullying. Rather than approaching victims and perpetrators as separate entities, these results support the importance of understanding the relationship dynamics between perpetrators and victims when developing intervention measures.

## Figures and Tables

**Table 1:** Participants' characteristics, personality traits, mentalization, organizational culture, and workplace bullying perpetration (*N* = 287).

Characteristics	Categories	*N* (%)	*M* (SD)	Skewness	Kurtosis
Sex	Female	270 (94.1)			
	Male	17 (5.9)			

Marital status	Single	181 (63.1)			
	Married	106 (36.9)			

Religion	No	156 (54.4)			
	Yes	131 (45.6)			

Educational level	3-year college	37 (12.9)			
	Bachelor's degree	225 (78.4)			
	≥ Master's degree	25 (8.7)			

Age (years)			32.45 (5.40)	1.14	1.48

Total working years			7.70 (4.30)	1.33	1.63

Position	Staff nurse	241 (84.0)			
	Charge nurse	46 (16.0)			

Subjective health status	Poor	48 (16.7)			
	Good	239 (83.3)			

Antibullying education	No	147 (51.2)			
	Yes	140 (48.8)			

Previous workplace bullying victimization	No	227 (78.1)			
	Yes	60 (20.9)			

Narcissistic grandiosity			33.64 (13.16)	−0.02	−0.13

Narcissistic vulnerability			40.36 (18.61)	0.20	−0.51

Perfectionistic self-presentation			84.16 (16.06)	−0.02	0.65

Mentalization			87.67 (10.64)	0.36	0.14

Organizational culture			86.68 (18.52)	−0.03	−0.07

Workplace bullying perpetration			38.40 (15.67)	1.07	0.34

*Note: M* = mean.

Abbreviation: SD = standard deviation.

**Table 2:** Comparison of workplace bullying perpetration by participants' characteristics (*N* = 287).

Characteristics	Categories	Workplace bullying perpetration		
		*M* (SD)	*t*/*F*	*p*
Sex	Female	40.40 (15.02)	0.56	0.575
	Male	38.27 (15.73)		

Marital status	Single	38.43 (15.83)	0.04	0.966
	Married	38.35 (15.46)		

Religion	No	35.52 (14.04)	3.47	0.001
	Yes	41.83 (16.83)		

Educational level	3-year college (a)	43.59 (18.11)	2.55	0.080
	Bachelor's degree (b)	37.43 (14.71)		
	Master's degree (c)	39.50 (19.05)		

Position	Staff nurse	38.54 (15.55)	0.34	0.732
	≥ Charge nurse	37.67 (16.46)		

Subjective health status	Poor	46.54 (17.96)	3.54	0.001
	Good	36.77 (14.67)		

Antibullying education	No	36.70 (14.11)	1.88	0.061
	Yes	40.19 (17.03)		

Previous workplace bullying victimization	No	35.52 (13.24)	5.27	< 0.001
	Yes	49.32 (19.13)		

*Note: M* = mean.

Abbreviation: SD = standard deviation.

**Table 3:** Correlation among variables (*N* = 287).

	Total working years	Narcissistic grandiosity	Narcissistic vulnerability	Perfectionistic self-presentation	Mentalization	Organizational culture	Workplace bullying perpetration
	*r* (*p*)	*r* (*p*)	*r* (*p*)	*r* (*p*)	*r* (*p*)	*r* (*p*)	*r* (*p*)
Total working years	1						
Narcissistic grandiosity	0.02 (0.778)	1					
Narcissistic vulnerability	0.05 (0.371)	0.80 (< 0.001)	1				
Perfectionistic self-presentation	0.03 (0.678)	0.49 (< 0.001)	0.52 (< 0.001)	1			
Mentalization	−0.04 (0.462)	−0.09 (0.116)	−0.29 (< 0.001)	0.06 (0.323)	1		
Organizational culture	0.05 (0.358)	−0.01 (0.848)	−0.15 (0.013)	0.03 (0.623)	0.27 (< 0.001)	1	
Workplace bullying perpetration	0.01 (0.824)	0.42 (< 0.001)	0.47 (< 0.001)	0.15 (0.012)	−0.31 (< 0.001)	−0.12 (0.040)	1

**Table 4:** Influencing factors of workplace bullying perpetration (*N* = 287).

Variables	*B*	SE	*β*	*t*	*p*	VIF	95% confidence interval	
							Lower	Upper
(Constant)	50.07	8.14		6.15	< 0.001		34.04	66.10

Religion^†^	1.63	1.62	0.05	1.01	0.316	1.09	−1.56	4.81
Health status^†^	3.11	2.30	0.07	1.35	0.178	1.23	−1.42	7.64
Prior workplace bullying victimization^†^	6.86	2.17	0.18	3.16	0.002	1.30	2.59	11.13
Narcissistic grandiosity	0.25	0.10	0.21	2.39	0.017	3.06	0.04	0.45

Narcissistic vulnerability	0.18	0.08	0.21	2.17	0.031	3.83	0.02	0.34
Perfectionistic self-presentation	−0.08	0.06	−0.09	1.42	0.156	1.49	−0.20	0.03
Mentalization	−0.28	0.08	−0.19	3.33	0.001	1.28	−0.44	−0.11
Organizational culture	0.02	0.05	0.02	0.34	0.734	1.18	−0.07	0.11

*Note:* Adjusted *R*^2^ = 30.1, *F* = 16.41, and *p* < 0.001. Durbin–Watson's *d* = 1.95 (du = 1.86, 4-du = 2.14) and Kolmogorov–Smirnov test (*Z* = 0.08, *p*=0.064).

Abbreviations: SE = standard error and VIF = variance inflation factor.

^†^Dummy variable (reference): religion (no), health status (good), and prior workplace bullying victimization (no).

## Data Availability

The data that support the findings of this study are available from the corresponding author upon reasonable request.
